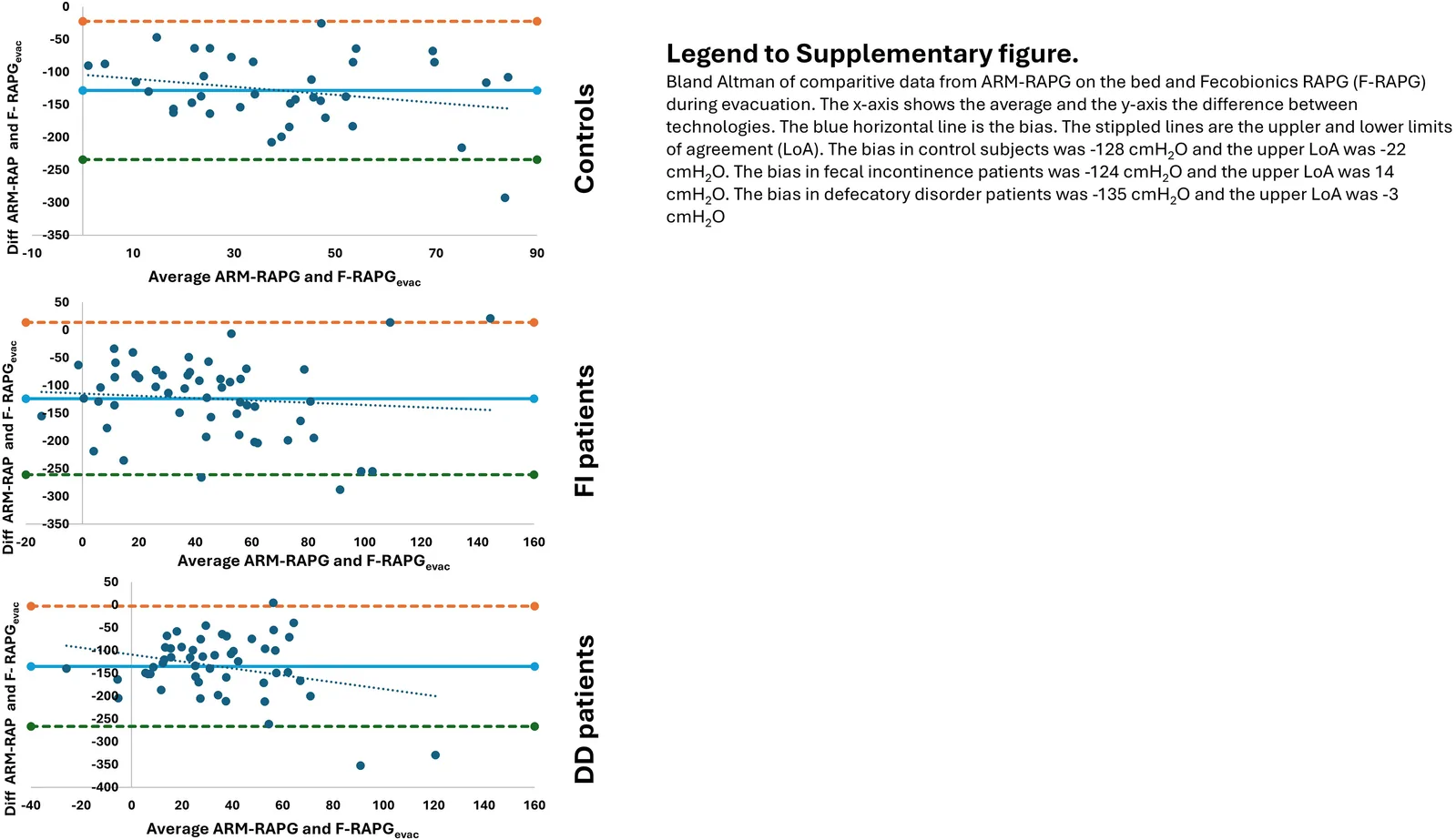# A Dual-Technology Multicenter Study of the Rectoanal Pressure Gradient as a Metric in Anorectal Pathophysiology

**DOI:** 10.1016/j.gastha.2026.101125

**Published:** 2026-10-09

**Authors:** Hans Gregersen, Giuliana Barron Del Solar, Xiaomei Guo, Danielle Long, Abeer Aziz, Delaram Asadi, Karlo Fidel, Samuel Eisenstein, Fouad J. Moawad, Satish SC. Rao

**Affiliations:** 1 GIOME, California Medical Innovations Institute, San Diego, California; 2 Department of Gastroenterology, J. WellStar MCG Health Augusta University, Augusta, Georgia; 3 Department of Surgery, University of California, San Diego, California; 4 Department of Gastroenterology, Scripps Clinic, San Diego, California

**Keywords:** Anorectal Physiology, Defecatory Disorder, Dyssynergia, Evacuation, Fecal Incontinence, Fecobionics, High-Resolution Anorectal Manometry

## Abstract

**Background and Aims:**

The rectoanal pressure gradient (RAPG) is a common metric for push maneuver in anorectal manometry (ARM). However, its artificial nature and negative values have been questioned. Fecobionics, a simulated stool, assesses anorectal pathophysiology more naturally, providing metrics including RAPG during evacuation. We compared the RAPG between ARM and Fecobionics in healthy participants (HP), and patients with fecal incontinence (FI) and defecatory disorder (DD) using Fecobionics as the dynamic evacuation-based comparator for ARM.

**Methods:**

Patients with FI (>1 episode/week) and DD (Rome IV criteria) and HP underwent push maneuvers during ARM in left lateral position and during Fecobionics in left lateral position and seated position (F-RAPG_push_) and during evacuation (F-RAPG_evac_). RAPGs including accumulated F-RAPG during the time course of the defecation attempt (F-RAPG_evac-accum_) were compared.

**Results:**

We evaluated 141 subjects, of whom 37 were HP (26%), 53 FI (38%), and 51 DD patients (36%). Fecobionics showed positive RAPG during push (∼15 cmH_2_O) and evacuation (∼100 cmH_2_O) compared to negative ARM-RAPG in all groups (∼−25 cmH_2_O, *P* < .001). Rectal pressure showed higher correlation with RAPG than anal pressure, indicating that rectal pressure is the most important determinant of RAPG. Most correlations were stronger for F-RAPG during push and evacuation compared to ARM-RAPG. DD had a stronger anal component than the other groups. The maximum ARM-RAPG and F-RAPG did not differ between groups, whereas F-RAPG_evac-accum_ differed between groups (*P* < .05).

**Conclusion:**

ARM-RAPG differed from F-RAPG and had low correlation with F-RAPG as well as with rectal and anal pressures. ARM-RAPG did not distinguish between groups. In contrast, F-RAPG_evac-accum_ distinguished between DD patients and the other 2 groups.

## Introduction

Anorectal disorders including fecal incontinence (FI) and defecatory disorders (DDs) such as dyssynergic defecation are highly prevalent conditions that significantly impair quality of life (QoL) and impose substantial personal and societal burden.^1^, ^2^, ^3^, ^4^ Because symptom patterns often overlap across different pathophysiological mechanisms, accurate physiological assessment is essential for both diagnosis and guiding targeted therapies.^2^, ^5^, ^6^, ^7^ Central to this assessment is the evaluation of rectal propulsion, anal sphincter relaxation, and their coordination during defecation, often summarized by the relationship between the rectal and anal pressures during attempted evacuation.

High-resolution anorectal manometry (ARM) is the most widely used technique to assess anorectal pressure profiles and coordination during rest, squeeze, and simulated defecation.^8^, ^9^, ^10^ ARM systems provide pressure topography and enable derivation of the rectoanal pressure differential (more commonly called the rectoanal pressure gradient [RAPG]), defined as the difference between rectal and anal pressures during attempted evacuation. It is commonly interpreted as an index of the driving force for defecation and adequacy of anal relaxation.^9^, ^10^ However, physiological and methodological limitations of ARM have been increasingly recognized. One of the most debated findings is the frequent observation of negative RAPG during simulated defecation, even in asymptomatic healthy participants (HP), a result that contradicts basic principles of fluid mechanics and expected pressure–flow relationships for evacuation.^11^, ^12^, ^13^ The unexpected negative gradients have raised concerns regarding the validity of RAPG as a surrogate for effective defecatory propulsion.^11^, ^12^

Additional limitations of ARM include the use of a stationary catheter and a rectal balloon that do not replicate the shape, compliance, weight, or movement of stool, potentially altering neuromuscular responses during attempted evacuation.^9^, ^11^, ^14^ Measurements may be influenced by catheter bending, axial motion, anal canal shortening, and changes in sensor orientation, particularly during straining.^8^, ^11^ Furthermore, the testing position, patient embarrassment, variable coaching, and reliance on voluntary simulation rather than actual evacuation can all affect the physiological relevance and reproducibility of ARM findings.^12^, ^15^ Collectively, these issues have led to calls for more physiologically representative assessment tools and for cautious interpretation of ARM–derived rectoanal pressure metrics in isolation.^11^, ^12^, ^13^, ^14^

Fecobionics is a novel device designed to simulate stool characteristics while simultaneously measuring axial intrarectal (rear) driving pressure and anal (front) resistive pressure during expulsion, allowing calculation of RAPG under more physiologic and dynamic conditions. By incorporating device deformation, movement, and expulsion through the anal canal, Fecobionics aims to better integrate rectal propulsion, anal opening, and outlet resistance into a single measurement paradigm.^14^, ^16^ While this approach introduces its own technical considerations—such as device compliance, friction, and expulsion mechanics—it offers a fundamentally different assessment of anorectal function compared with stationary ARM systems and may overcome some of the conceptual limitations associated with ARM–RAPG. Since Fecobionics assesses real evacuation, it is ideally suited to evaluate whether ARM-RAPG is physiologically sound, ie, serving as a dynamic evacuation-based comparator.

The aim of the present study is to compare the RAPG measured by Fecobionics during push procedures (F-RAPG_lying_ and F-RAPG_seated_), and during evacuation (F-RAPG_evac_) with the ARM-RAPG obtained during push simulated evacuation. By examining the agreement, systematic differences, and physiological correlations between these measures, this study seeks to determine whether they provide interchangeable or complementary information about anorectal function. Clarifying these relationships has the potential to improve interpretation of anorectal pressure metrics, address longstanding concerns regarding negative RAPGs, enhance pathophysiological phenotyping of DD and FI, and ultimately contribute to more precise diagnosis and individualized treatment selection.^2^, ^5^, ^11^, ^14^

## Methods

### Subjects

FI patients and DD patients (Rome IV criteria)^4^ and HP) were recruited at Augusta University in Georgia and the 3 San Diego based institutions University of California San Diego (UCSD), Scripps Clinic, and California Medical Innovations Institute. The subjects were recruited from January 2021 to October 2025. Patients with mixed symptoms of FI and dyssynergia^17^ were excluded to obtain more homogeneous patient groups. Patients with rectal prolapse and excessive perineal descent (>4 cm) were excluded. Patients with rectal mucosal intussusception and rectocele were, however, included in this group.

The inclusion criteria for HP and patients were 18 years of age or older with no upper age limit. For the HP group, if deemed “normal” during a phone interview where we asked about defecation patterns, medications, past medical history of GI surgeries and chronic diseases, subjects were invited to visit the anorectal function laboratory for further interview, questionnaires, and anorectal physiology testing. The HP group was carefully selected to avoid subjects who would masquerade anorectal symptoms. In addition to fulfilling the Rome IV symptom criteria,^4^ DD patients were required to have abnormal ARM. Additionally, when the balloon expulsion test was normal but ARM showed dyssynergia, these patients either had an abnormal colonic transit test or abnormal defecography or both in agreement with Rome IV. For all groups, data were obtained on gender, age, body mass index, symptoms, health status, disease conditions, and previous treatments. Attention was paid to past medical and surgical history (particularly noting any etiology of FI and DD) and obstetric history. For all groups, pregnant women were excluded. Fecal Incontinence Severity Index and FI QoL scores were obtained in FI patients and Patient Assessment of Constipation Quality of Life (PAC QoL) in DD patients.^18^, ^19^ After each of the ARM and Fecobionics tests, the subjects were asked about tolerability of the test. The tolerability score was a total of 10 questions each with a score between 1 and 5, ie, the total score ranging from 10 to 50 where higher numbers represent good tolerability.^20^ The questions covered the test procedures such as instructions, duration of the procedure, symptoms like pain, embarrassment, and unpleasantness with the studies. The study was done according to the Helsinki Protocol, institutional review board approved (Advarra Protocol IDs: Pro00052237 and Pro00052237A, and Augusta University institutional review board approval #1946573) and registered on www.clinicaltrials.gov with identifier NCT03317938. All authors had access to the study data and reviewed and approved the final article.

### Anorectal Manometry and Fecobionics Technologies

ARM was conducted with a standard high-resolution ARM system (Manoscan, Medtronic, Dublin, IE) and Fecobionics was done with the wireless insertable device (GI Bionics, San Diego) (Figure 1). ARM measures radial pressures during stationary placement of the catheter in anorectum, whereas Fecobionics measures axial pressures, ie, longitudinal in the direction of the defecatory trajectory before and during attempted evacuation of the device. The basic design of the wireless Fecobionics prototype has been previously described (Figure 1).^21^, ^22^, ^23^ In brief, the core of the insertable Fecobionics device was 10-mm OD and 10-cm-long with a 9 cm-long bag mounted. The core contains pressure sensors at the front, rear and inside the bag, motion processing units for computation of orientation and device bending, electrodes for impedance planimetric measurement of cross-sectional areas, and other electronic components (circuit boards, wireless transmitter, and batteries) embedded in the silicone core. Only pressure data from Fecobionics were analyzed in this study since ARM only measures pressure but not cross-sectional areas and bend angle. The cylindrical bag mounted on the core was used for rectal distension. With the architecture, hardness shore, and the bag, Fecobionics has the form and consistency that corresponds approximately to type 3 to 4 on the Bristol stool form scale.^24^ Types 3 to 4 are found in 60% of HP.^24^ The bag was connected through a detachable fill tube extending from the front of Fecobionics to a syringe containing body-warm saline. Wireless data transmission was done to a wireless receiver unit placed next to the subject in real-time using the ISM band (902–928 MHz).

**Figure 1:**
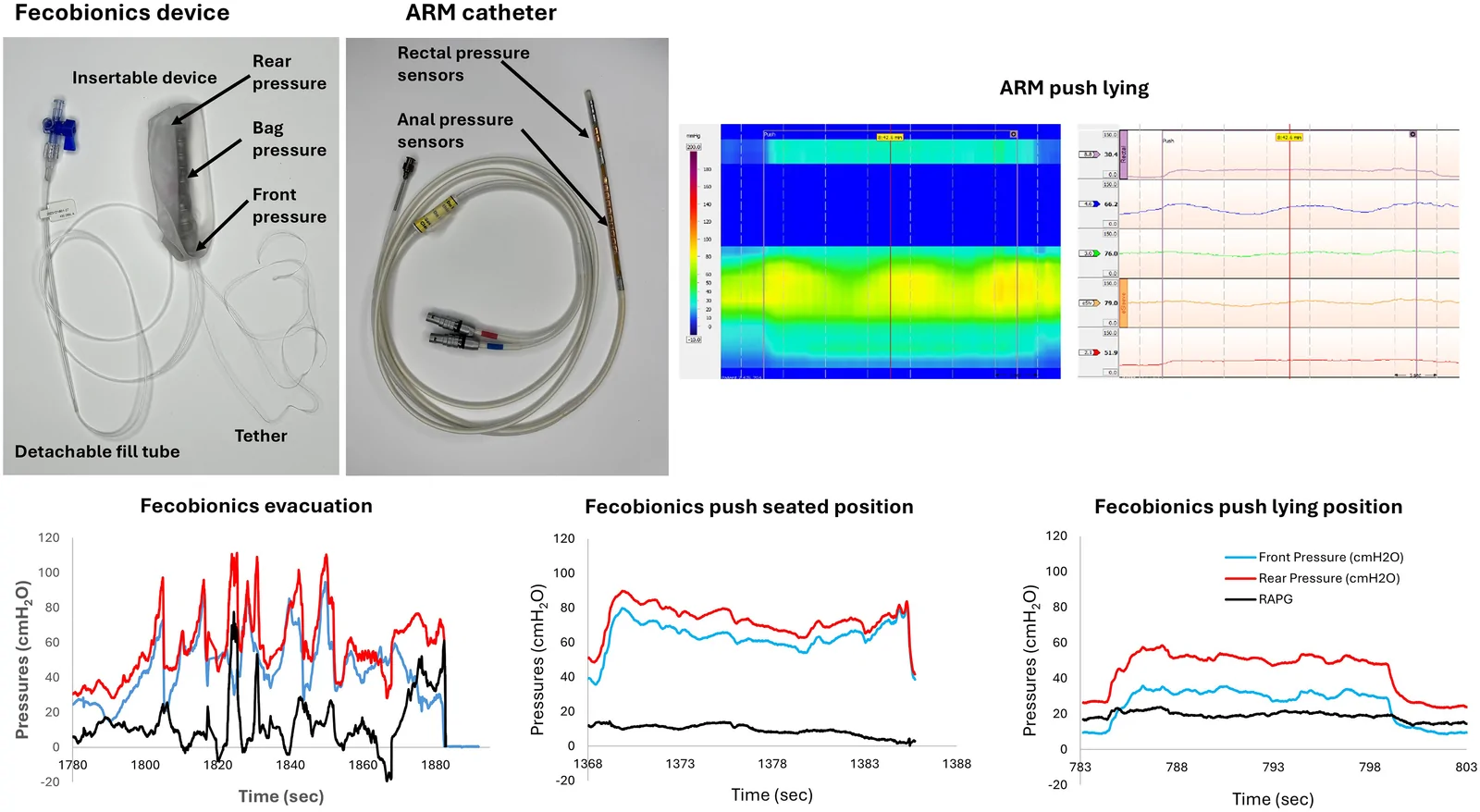
Upper left: Photos of the 2 devices used in the studies. The remaining panels show data from a typical patient with defecatory disorder. Upper right: Anorectal manometry (ARM) data presented as a color contour plot and tracings. Lower panels: Fecobionics evacuation and push in lying and seated positions. The black tracings are the rectoanal pressure gradient (RAPG). The pressure was highest during evacuation. The pressure during push in seated position was higher than in lying position.

### Procedures

Prior to testing, HP and patients were asked to empty their rectum. Enema was not used to simulate physiological conditions. Digital rectal examination was performed prior to insertion of Fecobionics or the ARM catheter to assess anal tone and verify that the lower rectum was empty. All experiments were performed in accordance with relevant guidelines and regulations.

ARM was conducted before or on the same day as the Fecobionics test. If done on the same day, the test order was randomized with at least 20 min between tests. The ARM catheter was inserted with the subjects lying in left lateral position with bent hips and knees. The catheter was placed in the rectum and pressure was measured at 0.5 cm distance in the anal canal and in rectum (Figure 1). The International Anorectal Physiology Working Group guidelines (the London protocol) were followed.^25^ For this study, primarily the push maneuver was of interest and analyzed. Push was done lying on the bed for ARM. balloon expusion test (BET) was done using a standard catheter (Mui Scientific) using a standard 50 mL volume.

Fecobionics was manually inserted in rectum with the subjects in left lateral position. A protocol aligned with the London protocol was followed with the subjects doing anal pull throughs, anal squeezes, 3 push maneuvers, straining, bag filling to urge sensation, and evacuation of the device. Data from other maneuvers than push, filling, and evacuation are not further described in this paper. The push maneuver was done the same way as with ARM in lying position on the bed as well as in seated position on the commode. Subsequently, the bag was filled with saline until the subjects reported urge-to-defecate sensation. The bag filling was stopped at the urge sensation level or when the bag was filled with a maximum volume of 100 mL, whatever came first. Hence, in contrast to BET that uses the same 50 mL volume for all, Fecobionics uses an individualized urge-controlled preload. The fill tube was detached after the urge sensation level was reached and the subject was allowed to evacuate the untethered device in privacy. All procedures after moving to the commode were conducted with a curtain shielding the subjects from the investigators and the investigators left the room during the final evacuation.

### Data Analysis

Two investigators independently evaluated the ARM and Fecobionics data while blinded to each other's results. A third investigator, who did not have a potential conflict of interest, did the statistical analysis independently. The primary outcomes were the F-RAPG_evac_ (considered the benchmark, ie, a dynamic evacuation-based comparator), ARM-RAPG, F-RAPG_push-seated_, and F-RAPG_push-lying_. ARM pressure data were converted from mmHg to cmH_2_O to make it comparable to Fecobionics data. Numerous other outcome parameters were analyzed too including anal and rectal pressure amplitudes from both devices, BET duration, Fecobionics expulsion duration, and questionnaire scores. Furthermore, the single-point F-RAPG_evac_ was accumulated (integrated RAPG over time of the evacuation attempt, ∫RAPGdt) and named the F-RAPG_evac-accum_ as well as a parameter that considered the urge-sensation volume was computed, ie, F-RAPG_evac-accum∗vol_. In these calculations both negative and positive RAPG values were considered. Recordings from subjects who could not evacuate the device were capped at 2 min. F-RAPG_evac-accum_ quantifies the total net propulsive effort put in by the subject to overcome anal resistance and friction. Factoring in the urge volume allows assessment of how efficiently a subject generates a pressure gradient relative to the functional load they are attempting to expel. Finally, to evaluate whether a higher accumulated RAPG was due to prolonged effort, a duration-normalized F-RAPG_evac-accum_ parameter was calculated that normalized the accumulated RAPG for evacuation duration.

The ARM-RAPG was measured by the Manoscan system as the best of the 2 to 3 push maneuvers. To be consistent, the same was done for Fecobionics push maneuvers. The ARM analysis followed the manufacturers’ settings where the software specifically looks for the most positive (or least negative) difference between the rectal and anal pressures over a specific window, typically a 3-second epoch within the maneuver.

Median and interquartile range (IQR), Kruskal-Wallis, Friedman, and Wilcoxon signed rank tests were used for statistical analysis of nonparametric nonpaired or paired data. Correlations were used for analysis of association of data obtained with the technologies employed and for the pressure components of RAPG. Bland-Altman plots were used to assess the agreement between the 2 measurement techniques. The bias (mean difference) and limits of agreement were computed. Results were considered statistically significant when *P* < .05 (2-tailed). Excel and SigmaStat were used for statistical testing.

## Results

### Entire Cohort and Major Group Data

One hundred forty-one subjects participated in the study, of whom 37 (26%) were HP (24 females [F]/13 males [M]), 53 (38%) were FI patients (41F/12M), and 51 (36%) DD patients (48F/3M). Table 1 provides demographic information. In brief, the subjects were 56 (45–62) years of age with the DD patients being marginally younger than FI patients. Most subjects were White and African American, and about 10% were other races. The body mass index was 27 (IQR 23–31) kg/m^2^ without difference between groups. Comorbidities and symptoms are listed in Table 1. The Fecal Incontinence Severity Index score and FI QoL score in FI patients were 28 (20–36) and 66 (49–86). The Constipation PAC QoL score in DD patients was 46 (30–72).

**Table 1:** Demographic Data

Demographic and clinical data	Control subjects	Fecal incontinence	Defecatory disorder
Age (y)	57 (46–66)	62 (50–70)	51 (40–64)
Sex (F/M) ratio%	24/13 (65%/35%)	41/12 (77%/23%)	48/3 (94%/6%)
Race	White 68% African American 16% Other 16%	White 51% African American 41% Other 8%	White 64% African American 26% Other 10%
BMI (kg/m^2^)	26 (23–31)	28 (24–33)	26 (22–31)
Obstetric profile and pregnancy	Pregnancy: 11/16 Vaginal delivery 8/11 C-section: 1/11 Mixed: 2/11 Hysterectomy: 3/16	Pregnancy: 33/37 Vaginal delivery 26/33 C-section: 4/33 Mixed: 3/33 Hysterectomy: 16/37	Pregnancy: 35/48 Vaginal delivery 22/35 C-section: 13/35 Hysterectomy: 16/48
Comorbidities	Diabetes mellitus: 1/19 Hypertension: 1/19 Hypercholesterolemia: 2/19	Diabetes mellitus: 7/53 Hypertension: 16/53 Hypercholesterolemia: 8/53 Gallbladder removed: 17/53 Chronic pain + meds: 8/53 Thyroid disease: 3/53	Diabetes mellitus: 2/51 Hypertension: 6/51 Hypercholesterolemia: 6/51 Gallbladder removal: 6/51 Chronic pain + meds: 16/51 Thyroid disease: 9/51
Back surgery or injury	Back surgery: 4/19	Back surgery: 14/37 Back injury: 13/37 Back pain: 2/37 Both (injury + surgery): 6/37	Back pain: 17/51 Back surgery: 1/51
Duration of fecal incontinence or constipation (y)		6 (2–17)	10 (5–26)
Type of leakage		Both (liquid-stool): 33/53 Stool: 9/53 Liquid: 11/53	
Number of bowel movements (BMs)		1 time/day: 10/46 1–3 times/day: 20/46 4–9 times/day: 15/46 10 or more/day: 1/46	1–2 times per 1–2 d: 11/51 2 times/week: 18/51 1 time/week: 7/51 Less than once/week: 12/51 Less than once/month: 3/51
Symptons associated — constipation			Abdominal pain: 11/51 Abdominal pain + feeling incomplete evacuation: 2/51 Feeling incomplete evacuation: 24/51
Assisted defecation			Laxative: 29/51 Laxative and enema: 18/51

Table shows demographic data for the 3 groups. Data are reported percentages or as median and quartiles (in parentheses).

BMI, body mass index; F, female; M, male.

### Basic Anorectal Manometry-BET and Fecobionics Data

The median and IQR maximum resting anal pressure on ARM was 95 (76–112), 92 (64–125), and 106 (76–127 cmH_2_O in HP, FI and DD patients, respectively (medians corresponding to 70, 67, and 78 mmHg). The anal squeeze pressure was 239 (167–281), 194 (129–249), and 203 (164–243) cmH_2_O in HP, FI, and DD patients, respectively (corresponding to 176, 143, and 149 mmHg). The anal squeeze pressure in FI patients was lower than in HP and DD (*P* < .05).

Twelve (34%) HP were unable to evacuate BET in 2 min, whereas 23 (66%) could evacuate it (2 were missing). Twenty-one (40%) FI patients were unable to evacuate BET in 2 min, whereas 32 (60%) evacuated it. Thirty (60%) DD patients were unable to evacuate the balloon on BET, whereas the other 20 patients had normal BET.

The evacuation time for Fecobionics was 14 (8–24), 24 (15–41), and 42 (19–120) sec for HP, FI, and DD patients, respectively. All HP evacuated the device in less than 2 min, whereas 5 FI (9%) and 15 DD (29%) patients could not evacuate the device. The maximum rectal (rear) pressure during evacuation was 116 (99–154), 119 (87–146), and 102 (80–133) cmH_2_O in HP, FI, and DD, respectively. The maximum anal (front) pressure was 111 (88–149), 109 (58–141), and 87 (70–134) cmH_2_O in HP, FI, and DD, respectively. The maximum anal pressure was usually recorded at the beginning of the attempt, ie, during the first defecatory contraction, whereas the rectal pressure maximum was found towards the end of the expulsion.

### Rectoanal Pressure Gradient

RAPG data for the dynamic evacuation-based comparator F-RAPG_evac_, vs push maneuvers using ARM in the seated and lying position are shown in Figure 2. RAPG was positive for Fecobionics during evacuation and negative for ARM for all 3 groups separately and combined. F-RAPG push_seated_ and F-RAPG push_lying_ were positive and did not differ from each other (*P* > .5). All other comparisons between tests were highly significant (Friedman Q > 29, Wilcoxon Z > −3, *P* < .01). It is notable that there was no difference between the groups for either F-RAPG_evac_ (H = 0.74, *P* > .5), F-RAPG_lying_ (H = 2.41, *P* > .3) or ARM-RAPG (H = 4.51, *P* > .10).

**Figure 2:**
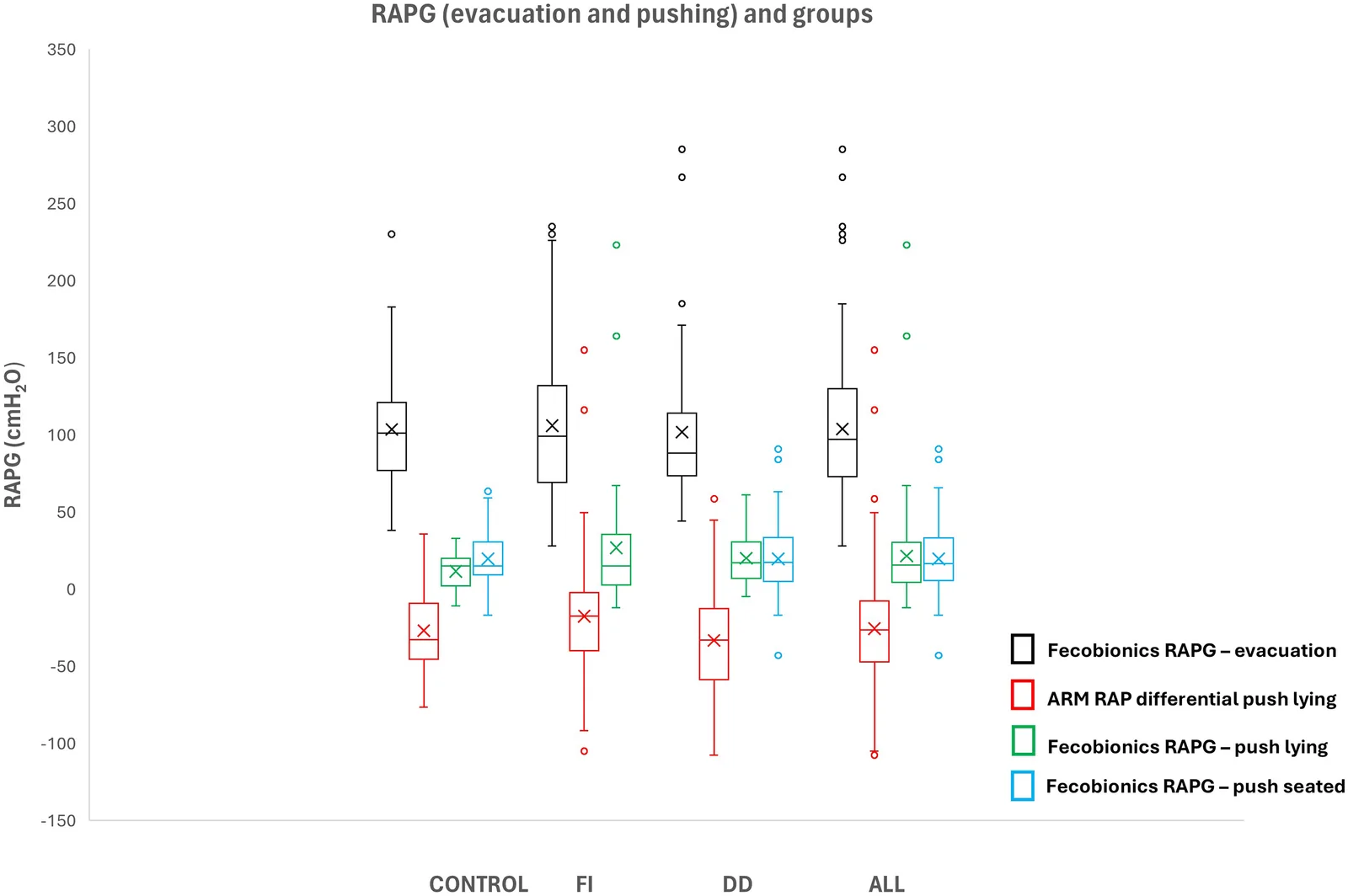
Box-Whisker plot of the rectoanal pressure gradient (RAPG) with the 4 maneuvers in the 3 groups for anorectal manometry (ARM) and Fecobionics. The boxes show the quartiles with average (the horizontal line and median (the cross). The whiskers show the maximum ex outliers. The dots are considered outliers. The maneuvers differed, whereas no difference was found between groups for either of the maneuvers.

For all subjects combined, no correlation was found between F-RAPG_evac_ and ARM-RAPG (Figure 3 left) and Bland-Altman plots showed significant RAPG bias between the 2 technologies (Figure 3 right). Furthermore, correlation between F-RAPG_evac_ and ARM-RAPG was not found in any single group (HP, FI, and DD). Bland-Altman plots showed significant bias for the HP and DD groups, whereas it was borderline for FI patients (Supplementary Figure).

**Figure 3:**
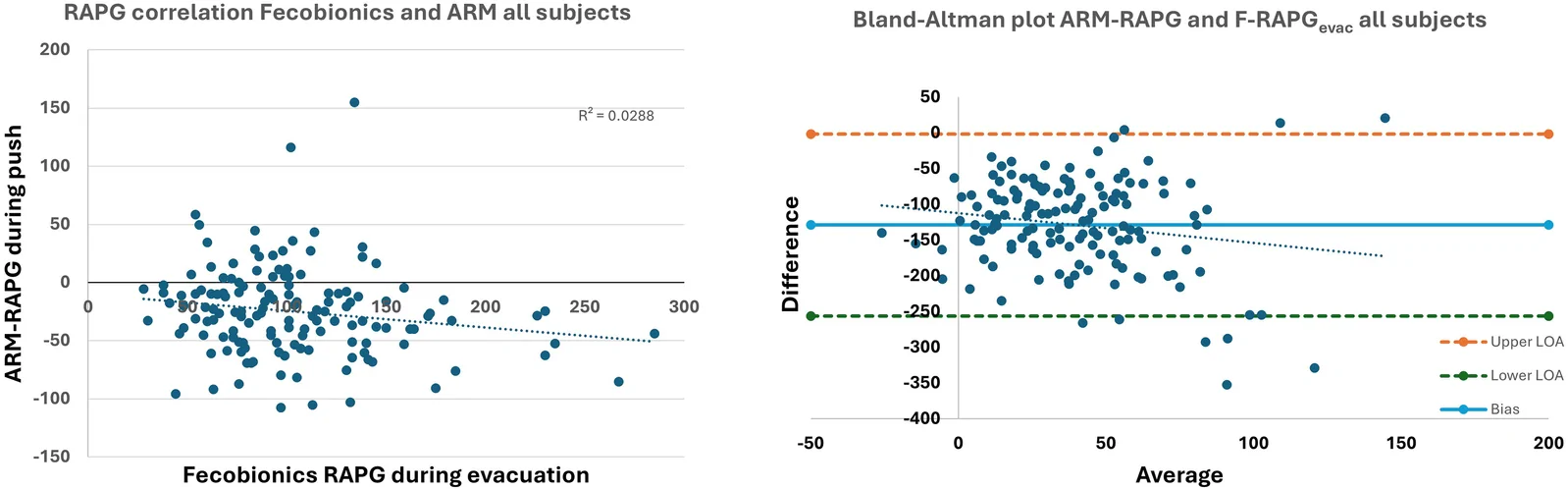
Correlation and Bland Altman plot for the rectoanal pressure gradient (RAPG) generated by Fecobionics during evacuation and anorectal manometry (ARM) during push. The 2 technologies provided significantly different RAPGs.

To better understand the difference between ARM-RAPG during push and F-RAPG_evac_, we evaluated the 2 components of RAPG, namely the rectal (driving) pressure and the anal (resistive) pressure in the 3 groups. The rectal and anal pressure components were plotted as function of RAPG (Figure 4).

**Figure 4:**
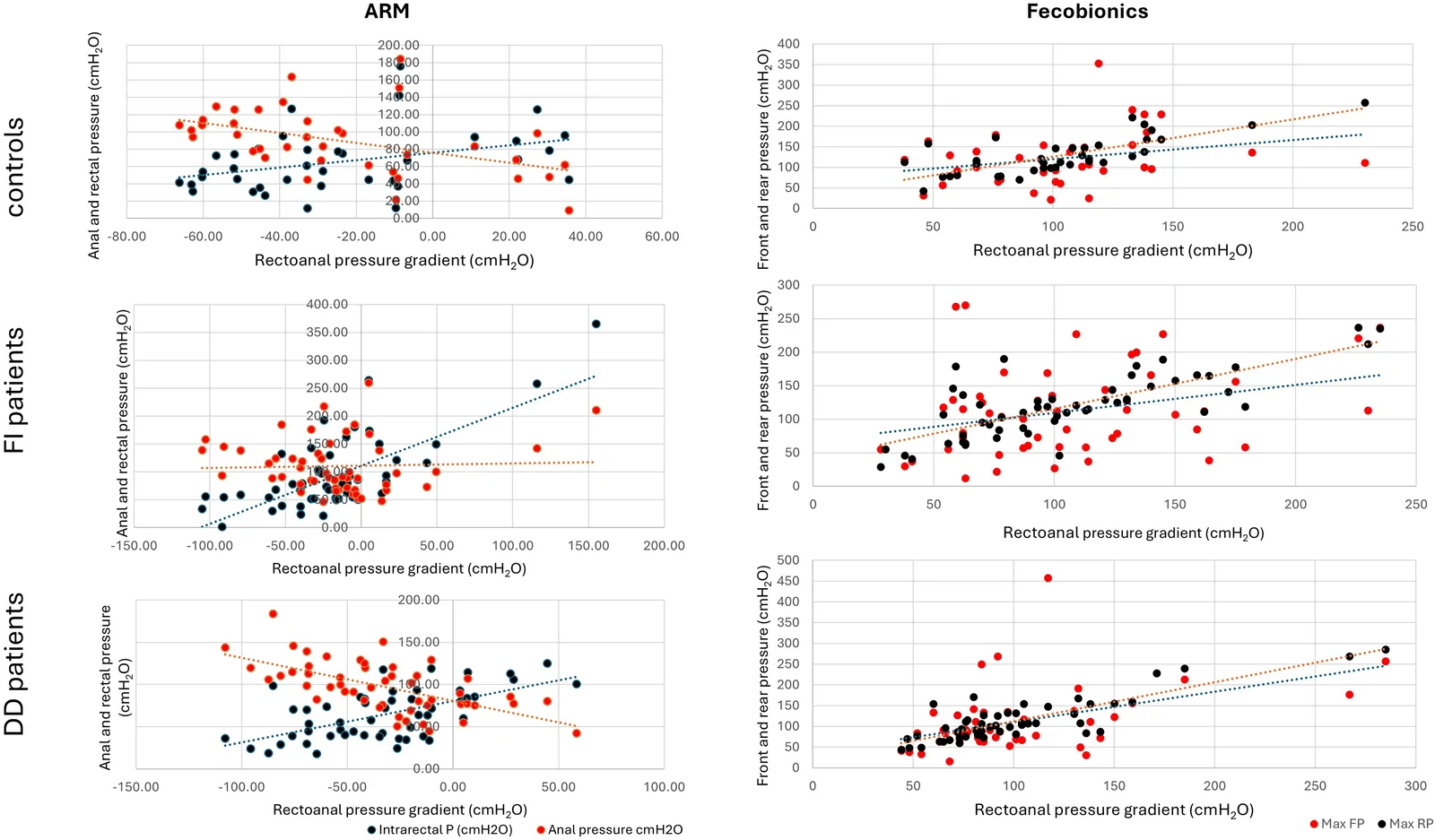
Correlation plots of ARM-RAPG and F-RAPG decomposed into the anal and rectal pressure components. In general correlations were strongest for Fecobionics. See text for explanations.

### Amorectal Manometry-Rectoanal Pressure Gradient (ARM-RAPG)

In HP, the correlation for anal pressure (r = 0.45) was marginally higher than for rectal pressure (r = 0.36), indicating that anal pressure is a slightly more important determinant of ARM-RAPG than rectal pressure. In FI patients, the rectal pressure (r = 0.70) was dominating over anal pressure (r = 0.03), indicating that the weak anal sphincter plays a minor role as predictor of RAPG. In DD patients, the anal pressure (R = 0.62) and rectal pressure (r = 0.60) were almost identical, indicating that the 2 components play an equal role. For the correlation plots for HP and DD, the slope of the anal regression line was negative and positive for the rectal pressure indicating that anal pressures are more important for low pressure defecators than for high pressure defecators, and vice versa.

### Fecobionics-Rectoanal Pressure Gradient During evacuation (F-RAPG_evac_)

In HP, the correlation for rectal pressure (r = 0.75) was higher than for anal pressure (r = 0.26). In FI, the rectal pressure (r = 0.76) was dominating over anal pressure (r = 0.32). In DD patients, the rectal pressure had a much higher correlation coefficient (r = 0.85) than anal pressure (r = 0.46), indicating that rectal pressure was most important. However, anal properties in DD played a bigger role than in HP and FI according to the magnitude of the correlations. Most correlations were stronger for Fecobionics RAPG compared to ARM-RAPG.

### Rao Dyssynergia Classification

Table 2 shows the distribution of types according to the Rao dyssynergia classification for HP and DD patients. The data were obtained in lying position. This analysis was not done for FI patients. The sensitivity of the Rao classification was high (45/51 abnormal types, 88%) but the specificity was low due to the many abnormal types in HP (14/37, 38%). Since the numbers are low for types 3 and 4, the following analysis only pertains to those with normal phenotype and type 1 and 2 in DD patients. No difference was found between normal and type 1 in the HP group for either Fecobionics or ARM, but F-RAPG was significantly higher than ARM-RAPG for both types. Likewise, no difference was found between normal, type 1, and type 2 in the DD group for either Fecobionics or ARM. F-RAPG was significantly higher than ARM-RAPG for both types.

**Table 2:** The Unit for all RAPG Data Is cmH_2_O, ie, ARM Data Were Converted From mmHg to cmH_2_O

Rao dyssynergia type (obtained on the bed)	Normal type	Type 1 (paradoxical contraction, adequate push)	Type 2 (paradoxical contraction, inadequate push)	Type 3 (no relaxation, adequate push)	Type 4 (no relaxation, inadequate push)	Unclassified
Controls	23/37	7/37	1/37	3/37	3/37	
Defecatory disorder (DD)	6/51	16/51	17/51	5/51	6/51	1/51
Controls ARM-RAPG (pressures)	−37 (−45 to −11)	−33 (−49 to −16)				
Controls F-RAPG_evac_	101 (68–136)	112 (98–114)				
DD ARM-RAPG (pressures)	−38 (−42 to −28), *P* = .15	−27 (−55 to 4)	−53 (−69 to −28)			
DD F-RAPG_evac_	70 (62–94), *P* = .19	95 (79–134)	91 (76–111)			

No differences were found between normal type and type 1 in controls using ARM or fecobionics (both *P* > .2). Likewise, no difference was found in patients between normal type, type 1, and type 2 for ARM or fecobionics (both *P* > .15). Fecobionics RAPG was significantly higher than ARM-RAPG for all comparisons (*P* < .001).

### Fecobionics Maximum Rectoanal Pressure Gradient vs Accumulated Rectoanal Pressure Gradient

Accumulated ARM data during push were not available but it can readily be extracted for Fecobionics. F-RAPG is computed as a single maximum value during push or evacuation and as mentioned above it did not differ between groups. Therefore, it was studied whether F-RAPG_evac_ accumulated over the duration of the expulsion (F-RAPG_evac-accum_) and F-RAPG_evac-accum_ multiplied by the volume at urge sensation (RAPG_evac-accum∗vol_ were superior to RAPGs based on the maximum value. F-RAPG_evac-accum_ and RAPG_evac-accum∗vol_ differed between groups (H = 6.8, *P* < .05 and H = 8.8, *P* < .01 with DD having higher values than for HP and FI. This effect was primarily due to the prolonged effort duration for DD patients since the duration-normalized F-RAPG_evac-accum_ parameter did not differ between groups. Despite the lack of significant difference between groups, it is noteworthy that the maximum RAPG was 13% lower in the DD group compared to the HP group, whereas the duration-normalized F-RAPG_evac-accum_ was 16% larger in the DD group compared to the HP group. This indicates an increased effort per unit time. RAPG and duration-normalized F-RAPG_evac-accum_ were higher in males compared to females (*P* < .05), whereas F-RAPG_evac-accum_ did not differ significantly (*P* > .3). None of the RAPG parameters showed age-dependency (r was near-zero, *P* > .10).

### Safety and Tolerability

No serious adverse events occurred with either ARM or Fecobionics. In all cases, the ARM catheter and Fecobionics device were safely inserted into the anorectum. The Fecobionics tolerability score was 43 (36–47), 41 (39–46), and 42 (39–47) in HP, FI and DD, respectively, without difference between groups (*P* > .5). The ARM tolerability score was 35 (31–42), 37 (32–44), and 38 (32–43) in HP, FI, and DD, respectively, without difference between groups (*P* > .5). Fecobionics was more tolerable than ARM in all groups (*P* < .001).

## Discussion

The study evaluated ARM-RAPG against the dynamic evacuation-based comparator Fecobionics RAPG during evacuation. Our main findings of this detailed RAPG study showed that Fecobionics consistently provided positive RAPG values for push maneuvers and evacuation, whereas ARM had negative RAPG values. These findings were consistent among HP, FI patients and DD patients. Furthermore, multiple correlation analyses and findings in subtypes of dyssynergic patients point to that single-point RAPG does not have clinical discriminatory value. In contrast, Fecobionics RAPG accumulated over the expulsion time differed between groups. Other measures such as anal resting and squeezes pressures and DI were largely as expected, eg, low squeeze pressure in FI patients.

Defecation is a complex physiological process.^26^, ^27^ The defecatory process must ideally be studied as physiologically as possible. Only 3 technologies can be considered dynamic defecatory tests, namely BET, defecography, and Fecobionics. However, BET and defecography do not measure anorectal pressures and hence cannot provide information on the driving and resistive forces involved in normal or abnormal evacuation. Furthermore, ARM, BET, and defecography frequently yield conflicting results for patients with pelvic floor dysfunction, and the specificity for ARM and BET is low especially in lying position but also when seated,^28^ which was also confirmed in this study. Differences between tests, low accuracy, and validity led to the concept that 2 of 3 tests must be positive to confirm DD diagnosis.^25^ The simulated stool Fecobionics offers a new paradigm for dynamic anorectal diagnostic testing. Therefore, Fecobionics served as the dynamic evacuation-based comparator in this study. Fecobionics has demonstrated data consistent with BET evacuation times and defecography metrics, such as anorectal angles and perineal descent.^29^, ^30^, ^31^ Furthermore, it exhibits a higher correlation with symptoms than ARM, as well as a greater predictive capability than ARM for biofeedback outcomes in FI patients.^32^, ^33^

RAPG is a relatively simple metric that from a biophysical point of view makes great sense. It is a proxy of the net propulsive force, ie, the driving force measured in rectum minus the resistive force of the anal sphincter apparatus. This study clearly shows that ARM-RAPG is a poor proxy of real defecation as assessed by Fecobionics. ARM-RAPG values were negative, and differences were not found between the 3 groups. The Bland Altman plots showed a horizontal trend, ie, no dependence on magnitude. There was, however, significant scatter, which means there is a consistent nonproportional bias, not related to the size of the measurement, with random differences (variability) around that constant bias. This suggests that ARM consistently underestimates Fecobionics, but the error magnitude does not change with the average value. Due to high variability as shown in the Bland Altman plots, it is impossible to adjust ARM-RAPG data to positive values using a correction factor.

A positive RAPG indicates a normal defecatory maneuver using the standard approach. However, when using ARM in lying position, this gradient is negative in a large proportion of HP as shown in this study as well as in multiple previous studies.^11^, ^12^, ^13^ It would have been expected that ARM-RAPG would be more negative in patients with low propulsion pressure or with paradoxical contraction or reduced anal relaxation. This was, however, not the case. This study suggests that Fecobionics provides better parameters, such as the F-RAPG_accum_ to discriminate between HP and DD as it more accurately mimics the physiological pattern during defecation. Furthermore, RAPG results are dependent on the position adopted during the defecation maneuver (lateral decubitus or sitting), and balloon inflation status.^28^, ^34^ Why the ARM-RAPG is negative in HP and in patients is unclear. Sauter and coworkers al hypothesized that simulated defecation may drive the recording catheter against the wall of the anal canal, producing contact pressure.^35^ Furthermore, ARM measures anal pressure across the whole anal high-pressure zone that includes puborectalis, internal anal sphincter (which cannot voluntarily relax as the relaxation is brought about in part by the rectoanal inhibitory reflex [RAIR]) and the external anal sphincter. This speaks in favor of using other measures such as the Defecation Index (DI).^29^, ^33^, ^36^ It is conceivable that the complete relaxation of the anal sphincter seen on ARM is an artifact caused by movement of the pressure sensors out of the anal sphincter during abdominal straining. In 1 study, a significant displacement of the catheter during simulated evacuation was observed in 55% of overall HP, which might tend to exaggerate anal relaxation during this maneuver.^35^ This does not affect high-resolution ARM as much because the topographic display makes it easier for the operator to minimize probe movement during the procedure.^37^ We suggest that the bending properties of the relatively stiff ARM catheter may play a significant role. Furthermore, the push procedure demonstrated in this study has limited clinical value due to the artificial testing conditions. Other ARM parameters, BET, defecography, Fecobionics, and clinical symptoms are more optimal for DD diagnostics.

As expected, and in contrast to ARM-RAPG, F-RAPG had positive values but did not differ between groups. This questions the validity of using a single maximum RAPG measure in clinical diagnostics. In contrast the accumulated RAPG showed higher values in DD patients than the other 2 groups although this effect was largely driven by the longer evacuation time. The accumulated RAPG and the patterns of RAPG changes appear to be more important than single-point maximum RAPG values. Future work must evaluate the demonstrated gender differences shown in this study and Fecobionics-defined phenotypes where at least 6 different DD subtypes have been identified previously by Fecobionics.^33^

### Limitations

It is a potential limitation that ARM RAPG data were only available in lying position, whereas we have data for Fecobionics during push in lying and seated position as well as during evacuation. Fecobionics did not show differences between push in lying and seated position, indicating the technology is not very sensitive to positional changes. However, that does not exclude dependency of ARM-RAPG on position. Studies suggest that fewer healthy controls has dyssynergic phenotypes when studied by ARM in seated position compared to lying position,^11^, ^28^ though this has been disputed by others.^38^, ^39^ Furthermore, the average RAPG appears to be slightly positive in seated position.^40^ In a subset of our HP and DD material, we have additional push data available for seated position. This showed that 70% of HP and 60% of DD had negative RAPG values (compared to 90% with negative values on the bed), the remaining were zero or slightly positive (unpublished data). Consequently, seated position would not have changed our conclusions. The often-negative or near-zero ARM-RAPG constitutes a problem for ARM technology, and it is our impression that most clinical laboratories using ARM still do push in lying position. It is significantly different from the Fecobionics dynamic evacuation-based comparator that showed significantly higher values.

The differences in RAPG between technologies raise the question whether RAPGs by the 2 technologies reflect the same organ behavior and whether they measure different physiological phenomena. ARM technology measures radial pressures by a catheter, whereas Fecobionics measures axial and bag pressures by an insertable tether-less device, ie, as simulated feces, whereas ARM simulates defecation. This is an important distinction. Other important differences are the different mechanical properties of the devices and urge-sensation based filling by Fecobionics. Furthermore, measurements by the 2 technologies cover different parts of the anal sphincter complex and there are differences in analysis, ie, how the RAPGs are computed. Nevertheless, this study follows the recommended analysis by the manufacturers and publications, which makes the comparison relevant. Despite the differences between technologies, eg, difference in RAPG and lack of correlations, there were also similarities, eg, neither technology showed difference between groups for the single-point maximum RAPG.

In this study, we excluded patients with mixed symptoms of FI and dyssynergic defecation to obtain more homogeneous groups. However, this cohort warrants dedicated attention in future research as a distinct patient population.^17^

An alternative RAPG parameter for clinical diagnostics is the DI, which quantifies the effectiveness of an attempted defecation maneuver. An ARM-based DI and multiple Fecobionics DIs have been presented in the literature.^29^, ^33^, ^36^ Fecobionics DIs encompass various combinations of pressure parameters, evacuation duration, and urge volume. Therefore, comparing RAPG with DI and other defecation metrics for both modalities in future studies is relevant to determine their diagnostic performance. Furthermore, it is also relevant to compare with MRI defecography that has shown superiority to ARM for DD diagnosis.^41^

## Conclusion

This study showed that Fecobionics provides different and potentially more physiologically representative dynamic information in HP and patients compared to ARM. Single-point (maximum) RAPG parameters by ARM or Fecobionics do not have discriminatory value between patient groups and HP. F-RAPG accumulated over the expulsion time differed between DD and other groups. Hence, RAPG data accumulated as well as deeper analysis of dynamic parameters and phenotyping based on pattern recognition will provide better diagnostic strength. These aspects need further study.

## Figures and Tables

**Supplementary Figure:**